# IL‐13 modulates ∆Np63 levels causing altered expression of barrier‐ and inflammation‐related molecules in human keratinocytes: A possible explanation for chronicity of atopic dermatitis

**DOI:** 10.1002/iid3.427

**Published:** 2021-04-01

**Authors:** Terufumi Kubo, Sayuri Sato, Tokimasa Hida, Tomoyuki Minowa, Yoshihiko Hirohashi, Tomohide Tsukahara, Takayuki Kanaseki, Kenji Murata, Hisashi Uhara, Toshihiko Torigoe

**Affiliations:** ^1^ Department of Pathology, School of Medicine Sapporo Medical University Sapporo Japan; ^2^ Department of Dermatology, School of Medicine Sapporo Medical University Sapporo Japan

**Keywords:** atopic dermatitis, barrier, IL‐13, keratinocyte, type 2 inflammation, ΔNp63

## Abstract

**Background:**

Barrier disruption and an excessive immune response in keratinocytes are now considered to have important roles in the pathophysiology of atopic dermatitis (AD). Furthermore, disturbed keratinocyte differentiation is considered to underlie AD. ΔNp63, a p53‐like transcription factor, is a major regulator of keratinocyte differentiation. However, the functional significance of ΔNp63 in AD has not been clarified.

**Objective:**

In this study, we aimed to investigate the influence of the type 2 inflammatory environment on ΔNp63 expression and AD‐associated molecules regulated by ΔNp63 in keratinocytes.

**Methods:**

The immunohistochemical expression profiles of ΔNp63 and AD‐related molecules were evaluated in human skin tissue. The function of ΔNp63 in the regulation of AD‐related molecules and the influence of the type 2 inflammatory environment on ΔNp63 expression were investigated using human primary keratinocytes. Expression of ΔNp63 was manipulated using the RNA interfering method.

**Results:**

In healthy skin tissue, we observed an inverse expression pattern between ∆Np63 and some barrier‐related proteins including filaggrin, caspase‐14, claudin‐1, and claudin‐4. ΔNp63 regulated expression of these genes and proteins. In addition, production of IL‐1β and IL‐33, pro‐inflammatory cytokines, was modulated by ΔNp63. Furthermore, prolonged IL‐13 exposure increased the thickness of the three‐dimensional culture of keratinocytes. IL‐13 interfered with ΔNp63 downregulation during calcium‐induced keratinocyte differentiation. IL‐13 modulated some barrier‐related and inflammation‐related molecules, which were regulated by ΔNp63.

**Conclusions:**

We have shown that ΔNp63 modulated AD‐related barrier and inflammatory molecules. In addition, ΔNp63 expression was affected by IL‐4/IL‐13. IL‐13–ΔNp63 axis would integrate two major factors of AD pathogenesis: dysregulated barrier and inflammation.

## Graphical abstract

IL‐13 exposure inhibits keratinocyte differentiation by interrupting ΔNp63 downregulation. Decreased filaggrin and caspase‐14 expression further disturb epidermal barrier integrity, whereas increased IL‐1β and IL‐33 enhance inflammation. This IL‐13–ΔNp63 axis may constitute a vicious cycle, partly explaining the chronicity of atopic dermatitis.

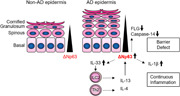

## 1 INTRODUCTION

Atopic dermatitis (AD) is a chronic inflammatory skin disorder and its prevalence is about 8%–13% and 2.5%–9.4% in Japanese children and adults, respectively.^1^ Recurrent eczematous lesions and intense itching caused by AD often cause sleep disturbance, which may profoundly impair patients' quality of life.^2^ Furthermore, accumulating epidemiological and mechanistic evidence has revealed that bronchial asthma or food allergy, which are occasionally life threating, may be attributable to AD.^3^, ^4^ Thus, appropriate treatment and primary prevention of AD are worldwide concerns. Although the recently developed drug dupilumab, which is an antibody that blocks interleukin (IL)‐4 receptor α, is effective for the treatment of severe AD, it is expensive.^5^ Therefore, dupilumab may not be suitable for every AD patient. It is important to understand the landscape of AD pathogenesis for primary prevention as well as radical treatment.

Physiologically, the keratinocyte provides the primary physical and immunological barrier preventing invasion. Accumulating evidence has revealed that dysfunction of the epidermal barrier and an overactive immune response underlie AD pathogenesis.^6^ Filaggrin and caspase‐14, a filaggrin‐cleaving enzyme, are reduced in AD.^7^, ^8^, ^9^ In addition, attenuated expression of claudin‐1, a tight junction protein, has been identified histologically.^10^, ^11^ Furthermore, decreased claudin‐1 promotes AD‐like skin inflammation in a mouse model.^12^ Classically, AD has been considered antigen‐specific inflammation caused by type 2 helper T (Th2) cells and IgE‐mediated mast cell degranulation. However, recent studies have revealed that antigen‐independent type 2 innate lymphoid cells (ILC2) play a crucial role in the inflammatory environment.^13^ In response to stimulation of endogenous or exogenous agents, keratinocytes release abundant epithelium‐derived cytokines, such as thymic stromal lymphopoietin (TSLP) and IL‐33, which induce and activate ILC2 and Th2 cells. In addition, IL‐4 and IL‐13, which are type 2 inflammation‐related cytokines, further disturb the epidermal barrier integrity.^14^ This vicious cycle is proposed to be the main cause of the chronicity of AD. Keratinocytes have a primary role in AD pathophysiology. Notably, dysregulated keratinocyte differentiation underlies AD, although the detailed functional significance remains unclear.

p63 and p73 belong to the p53 family of transcription factors, which are preferentially expressed in various types of epithelium.^15^ Although highly conserved sequences are shared among these proteins, they have roles in determining proliferation, apoptosis, or differentiation in the context of carcinogenesis or development. p63 consists of two major isoforms, TAp63 and ΔNp63. TAp63 is strongly expressed in the oocyte, whereas ΔNp63 is the predominant isoform in squamous epithelium, including epidermal keratinocytes and the basal layer of the respiratory epithelium. Animal experiments revealed that the ratio TAp63/ΔNp63 seems to be involved in the appropriate differentiation of keratinocyte and development of epidermis, nevertheless further investigation is required.^16^ In a mouse model of skin‐specific ΔNp63 knockdown, epidermal erosion and disturbed wound healing were observed.^17^ These findings indicate that ΔNp63 is a crucial factor regulating epidermal morphogenesis and keratinocyte differentiation.^18^ In addition to these functions, we speculate that p63 and p73 are involved in the pathogenesis of AD or bronchial asthma by regulating barrier and immune function of epithelial cell.^19^, ^20^, ^21^

Therefore, we hypothesized that dysregulation of ΔNp63 expression in keratinocytes would lead to prolonged dysfunction of the epidermal barrier and overactivation of type 2 immunity. In this study, we aimed to investigate the influence of the type 2 inflammatory environment on ΔNp63 expression and AD‐associated molecules regulated by ΔNp63 using human primary keratinocytes.

## 2 MATERIALS AND METHODS

### 2.1 Tissues

Skin tissues without inflammation were obtained from Sapporo Medical University Hospital. Donors were a 3‐year‐old girl, a 32‐year‐old man, and a 50‐year‐old woman. All tissues were obtained with written informed consent according to the guidelines of the Declaration of Helsinki and with approval of the Institutional Review Board (IRB) of Sapporo Medical University Hospital (permit number 292‐137; entitled “protein expression analysis in normal skin tissue”).

### 2.2 Cell culture and stimulation

Normal human epidermal keratinocytes (NHEKs) were purchased from Kurabo Industries Ltd. NHEKs were derived from the forehead skin of two Caucasian male donors under the age of 1 year. NHEKs derived from a 3‐year‐old female Japanese donor were isolated in our laboratory.^22^ The cells were cultured as a monolayer in keratinocyte‐SFM supplemented with 5 ng/ml human recombinant epidermal growth factor 1–53 and 50 μg/ml bovine pituitary extract (Gibco) at 37°C in a humidified atmosphere with 5% CO_2_. NHEKs were maintained for three or four passages in these conditions.

We defined the pre‐differentiation stage as a semiconfluent monolayer of keratinocytes cultured in keratinocyte‐SFM (low calcium). Keratinocyte differentiation was induced by calcium supplementation (1.0 mM).^23^ The differentiating and differentiated stages were designated as NHEKs cultured in the differentiation conditions for 3 and 7 days, respectively. To develop culture of NHEKs, cells were seeded at a density of 5.0 × 10^5^ cells in a 12‐mm‐diameter polyester membrane with a pore size of 0.4 μm (Corning Costar) in the three‐dimensional (3D) culture medium (keratinocyte‐SFM:Dulbecco's modified Eagle's medium, 1:1) as previously reported.^24^ After the cells had grown to complete confluence, medium in the apical compartment was removed to allow the cells to differentiate. NHEK stimulation was initiated when the apical medium was removed.

For cell stimulation, culture medium was supplemented with polyinosine–polycytidylic acid (poly I:C; Novus Biologicals), IL‐4 (PeproTech), IL‐13 (PeproTech), and/or IL‐22 (PeproTech). The concentrations of each reagents are described in the figures or figure legends.

### 2.3 Small interfering RNA (siRNA) transfection

Human *TP63* (ΔNp63)‐specific siRNA was obtained from Invitrogen (Carlsbad; sense: 5ʹ‐ACAAUGCCCAGACUCAAUU‐3ʹ; antisense: 5ʹ‐AAUUGAGUCUGGGCAUUGU‐3ʹ). Scrambled sequence siRNA for the negative control was purchased from Invitrogen. Transfections were performed using Lipofectamine RNAiMAX (Invitrogen) in Opti‐MEM (GIBCO) at 40 nmol/L according to the manufacturer's instructions. The culture medium was replaced at 6 h after siRNA transfection. Keratinocyte used in siRNA transfection experiment was at proliferation phase.

### 2.4 Complementary DNA (cDNA) microarray analysis

Messenger RNA (mRNA) was extracted from NHEKs transfected with *TP63* (ΔNp63)‐specific or scramble sequence siRNA at 72 h after transfection. Microarray slides were scanned using a 3D‐GENE human 25k (TORAY) and microarray images were automatically analyzed with AROSTM, version 4.0 (Operon Biotechnologies). The result has been deposited in NCBIs Gene Expression Omnibus (GEO; http://www.ncbi.nlm.nih.gov/geo/) and are accessible through GEO Series accession number GSE162900.

### 2.5 Antibodies

The following antibodies were used: mouse anti‐p63 monoclonal antibody (mAb) (clone: 4A4; Abcam), mouse anti‐ΔNp63 mAb (clone: BC28; Biocare Medical), mouse anti‐β‐actin mAb (clone: AC‐15; Sigma‐Aldrich), mouse anti‐claudin‐4 mAb (clone: 3E2C1; Invitrogen), mouse anti‐filaggrin mAb (clone: AKH1; Santa Cruz Biotechnology), mouse anti‐caspase‐14 mAb (clone: EPR12927; Abcam), mouse anti‐Ki67 mAb (clone MIB‐1; Dako), rabbit anti‐claudin‐1 mAb (clone: EPRR18871; Abcam), and rabbit anti‐IL‐33 mAb (clone: EPR20417; Abcam). Peroxidase‐conjugated goat anti‐mouse and anti‐rabbit IgGs were purchased from Kirkegaard & Perry Lab Inc.

### 2.6 Polymerase chain reaction (PCR)

Total NHEK RNA was purified using RNeasy Mini Kit and RNase‐free DNase (Qiagen) according to the manufacturer's protocol. Extracted total RNA was reverse‐transcribed into cDNA using a RevertAid RT Kit containing random hexamer primers (Thermo Fisher Scientific). Quantitative PCR was performed with target gene‐specific primers (Invitrogen) and SYBR green PCR master mix (Thermo Fisher Scientific) using a QuantStudio 3 Real‐Time PCR System (Applied Biosystems) according to the manufacturer's instructions. Target gene expression was detected with primers (see Table S1). The amount of mRNA of elongation factor 1α was used to standardize the quantities of each transcript. The $2^{-∆∆C_{t}}$ method was used to calculate the relative gene expression level of triplicate specimens.

### 2.7 Immunohistochemical analysis

Immunohistochemical procedures were performed as previously described.^21^ Briefly, sections of formalin‐fixed paraffin‐embedded skin tissue or 3D‐cultured NHEKs were immunostained using mAbs after epitope retrieval by Target Retrieval Solution pH 9 (Dako). ΔNp63, filaggrin, caspase‐14, claudin‐1, claudin‐4, and Ki‐67 proteins were detected using specific antibodies at the concentrations recommended by the manufacturers.

### 2.8 Western blot analysis

Cultured NHEKs were lysed with radioimmunoprecipitation assay buffer containing protease inhibitors (Roche) for 30 min on ice. Aliquots of the supernatants under reducing conditions were applied to SDS‐5%–20% polyacrylamide gels (SuperSep™; FUJIFILM Wako Pure Chemical) and transferred onto polyvinylidene fluoride membranes (Millipore). The membranes were incubated with optimally diluted antibodies for 1 h at room temperature. After washing, the membranes were reacted with a peroxidase‐labeled secondary antibody for 45 min. Signals were visualized using an enhanced chemiluminescence detection system (Amersham Life Science). The levels of intensity of signals detected in immunoblots were quantified using NIH ImageJ software and standardized to the corresponding levels of β‐actin.

### 2.9 Enzyme‐linked immunosorbent assay (ELISA)

Cells were cultured at a density of 3 × 10^5^ cells/well in a 12‐well plate. Culture supernatants were collected at 48 h after the start of the experiment and analyzed in triplicate. Protein concentrations were determined with Human DUOset ELISA Kit (R&D Systems) for IL‐1β according to the manufacturer's instructions. Absorbance was measured using an iMark microplate absorbance reader (Bio‐Rad).

### 2.10 Statistical analysis

Data analysis was performed with Prism Version 6 software (GraphPad Software). Statistical significance was evaluated using the two‐tailed unpaired Student's *t* test. Results are presented as mean ± *SD*; each set of results shown is representative of at least three separate experiments.

## 3 RESULTS

### 3.1 Distribution of ∆Np63 and AD‐related barrier proteins in healthy human epidermis

**Figure 1:**
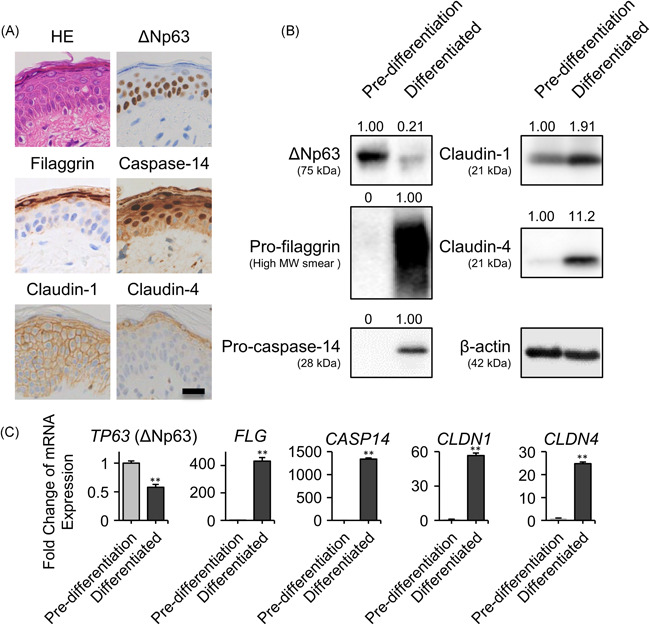
Expression profile of barrier‐related proteins at various stages of differentiation of human epidermal keratinocytes. (A) Histological expression pattern of ΔNp63, filaggrin, caspase‐14, claudin‐1, and claudin‐4. Scale bar = 50 μm.(B) Protein expression and (C) gene expression of *ΔNp63* (*TP63*), pro‐filaggrin (*FLG*), pro‐caspase‐14 (*CASP14*), claudin‐1 (*CLDN1*), and claudin‐4 (*CLDN4*) in proliferating or differentiated NHEKs. **p < .01.The data shown are representative of three independent experiments from three different donors. mRNA, messenger RNA; NHEK, normal human epidermal keratinocyte; HE, hematoxylin and eosin stain [Color figure can be viewed at wileyonlinelibrary.com]

To investigate the expression profile of ∆Np63 and AD‐related barrier proteins, we examined the histopathology of skin tissues unaffected by inflammatory skin diseases, including AD (Figure 1A). ∆Np63 was expressed in the basal to spinous layers, whereas ∆Np63 expression was lost in the granular layer. In contrast, expression of claudin‐4 and filaggrin was limited to the granular layer. Although claudin‐1 and caspase‐14 were detected in whole epidermis, their expression levels were slightly higher in the upper spinous layer compared with the basal layer.

Thus, we investigated the expression of these proteins in the cultured primary NHEK monolayer. As described above, NHEKs were cultured under two different conditions: “pre‐differentiated” and “differentiated” conditions are models of basal and apical side keratinocytes, respectively. In the Western blot analysis, ∆Np63 expression level was higher in pre‐differentiated keratinocytes compared with differentiated keratinocytes. In contrast, the barrier proteins claudin‐1, claudin‐4, pro‐filaggrin, and pro‐caspase‐14 showed an inverse pattern compared with ∆Np63 (Figure 1B; mature filaggrin or caspase‐14 were not detected in the two‐dimensional culture system). These results were also confirmed by evaluating mRNA expression levels (Figure 1C).

### 3.2 ∆Np63 controls expression of barrier‐ and inflammation‐related proteins

**Figure 2:**
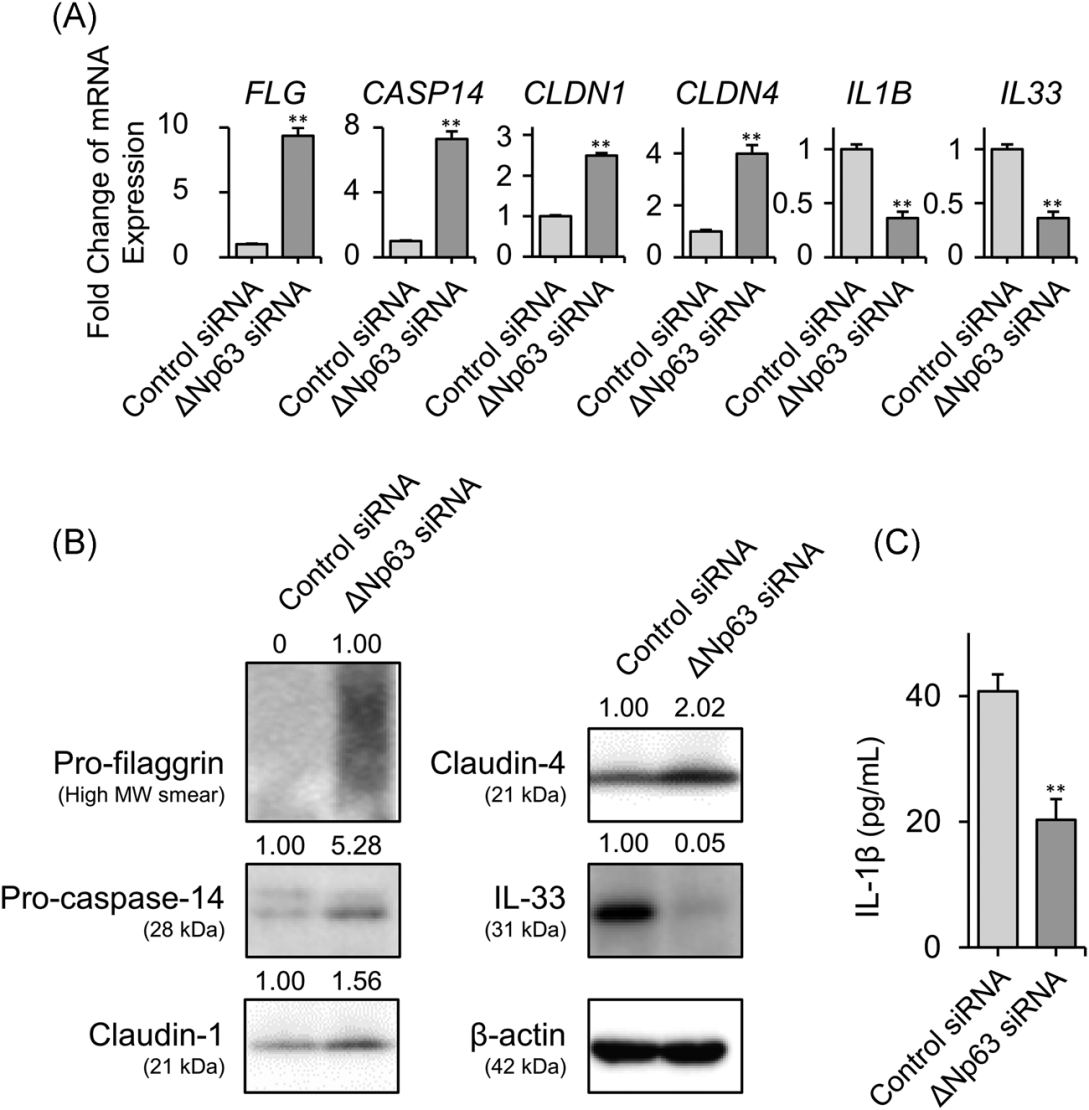
Modulation of AD‐related molecules by ΔNp63 in NHEKs. (A) Transcriptional expression of *FLG*, *CASP14*, *CLDN1*, *CLDN4*, *IL1B*, and *IL33* in ΔNp63‐knockdown and control NHEKs. (B) Protein expression of pro‐filaggrin, pro‐caspase‐14, claudin‐1, claudin‐4, and IL‐33 in ΔNp63‐knockdown and control NHEKs. Cells were harvested 72 h after transfection (A, B). (C) Protein levels of endogenous IL‐1β in the culture supernatant of *TP63* (ΔNp63)‐siRNA‐transfected and control keratinocytes in response to poly (I:C) stimulation. **p < .01. The data shown are representative of three independent experiments from three different donors. AD, atopic dermatitis; mRNA, messenger RNA; NHEK, normal human epidermal keratinocyte; siRNA, small interfering RNA

Based on the abovementioned inverse expression patterns between ∆Np63 and some barrier proteins, we hypothesized that ∆Np63 affects the expression of AD‐related proteins. Indeed, we previously reported that expression levels of claudin‐4 and TSLP receptor were modulated by ∆Np63 in HaCaT keratinocytes.^19^, ^25^ In this study, NHEKs expressing abundant ΔNp63 were transfected with *TP63* (ΔNp63)‐specific siRNA, which successfully downregulated ΔNp63 expression at the transcript and protein levels (Figure S1A,B). We could not find significant morphological alteration of the keratinocyte between these conditions. To investigate the transcriptional target of ΔNp63 in NHEKs, *TP63* (ΔNp63) or control siRNA‐transfected NHEKs were subjected to cDNA microarray expression analysis. As shown in Tables S2 and S3, ΔNp63 downregulated or upregulated approximately 1200 transcriptional gene expression levels by more than twofold. Interestingly, ΔNp63 negatively regulated the expression of the barrier‐related molecules *CASP14*, *CLDN1*, and *CLDN4*, which encode caspase‐14, claudin‐1, and claudin‐4, respectively. In addition to barrier‐related genes, ΔNp63 positively regulated *IL1B*, which encodes the pleiotropic pro‐inflammatory cytokine IL‐1β. We then confirmed the expression of these genes by quantitative PCR (Figure 2A). Gene expression of *FLG* and *IL33*, which encode filaggrin and IL‐33, respectively, were also modulated by ΔNp63; however, they were not detected in the microarray analysis (Figure 2A). At the protein level, expression of pro‐filaggrin, pro‐caspase‐14, claudin‐1, and claudin‐4 were negatively modulated by ΔNp63, whereas ΔNp63 positively regulated IL‐33 in the Western blot analysis (Figure 2B). According to a previous study, IL‐1β release from NHEKs is induced by poly (I:C) stimulation.^26^ As expected, ΔNp63 knockdown NHEKs released less IL‐1β upon poly (I:C) stimulation (Figure 2C).

### 3.3 IL‐13 inhibited downregulation of ∆Np63 expression during differentiation of keratinocytes

**Figure 3:**
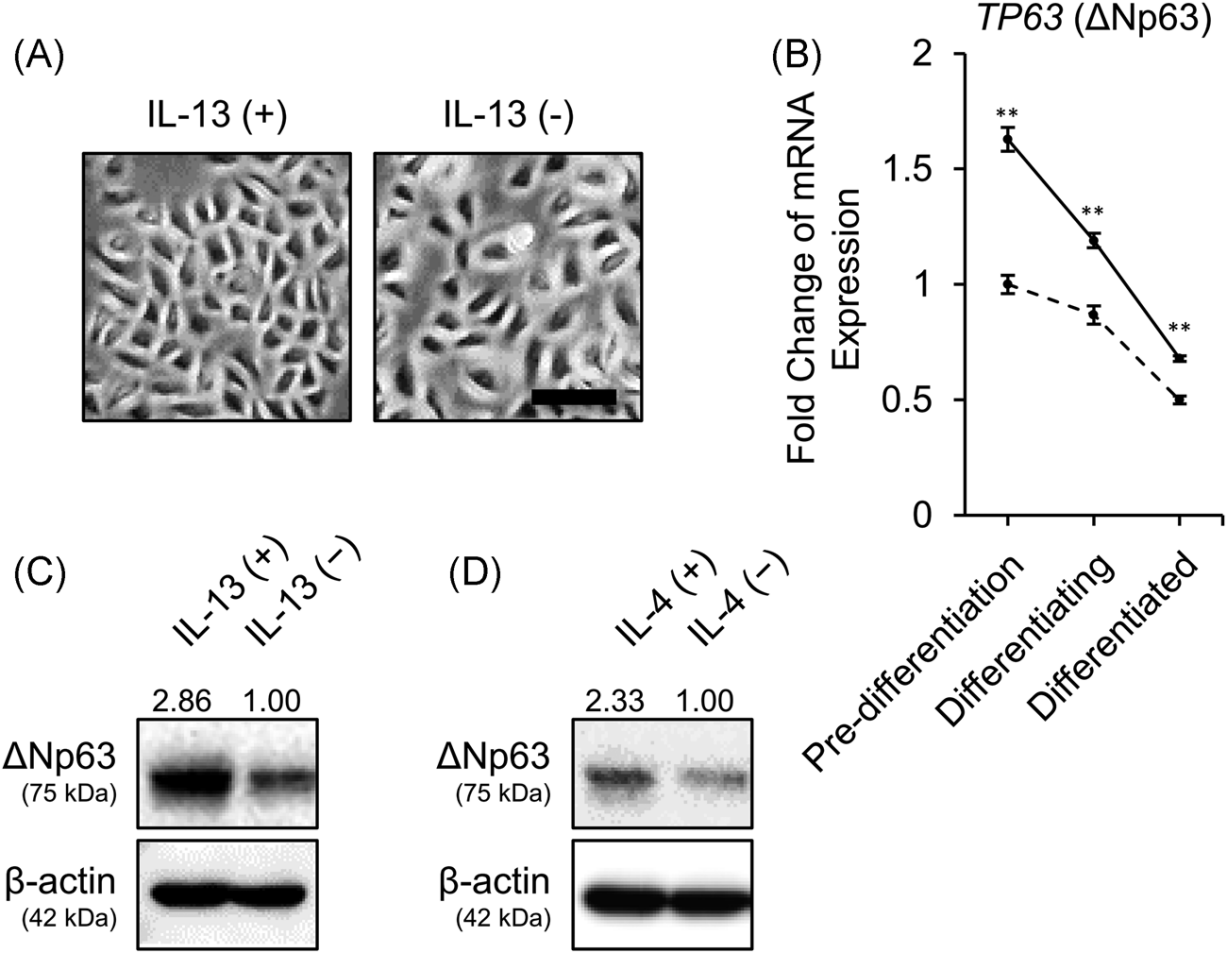
IL‐4/IL‐13 stimulation inhibits ΔNp63 downregulation during keratinocyte differentiation. (A) Phase‐contrast microscopy demonstrating the morphological changes of NHEKs at 48 h after treatment with 50 ng/ml IL‐13. Scale bar = 100 μm.(B) *TP63* (ΔNp63) gene expression in response to 50 ng/ml IL‐13 at various differentiation stages. NHEKs at the pre‐differentiation stage were cultured in low calcium medium. Differentiating and differentiated NHEKs were cultured in 1 mM calcium medium for 3 and 7 days, respectively. All NHEKs were stimulated for 24 h. **p < .01. (C, D) Alteration of ΔNp63 protein expression in response to 50 ng/ml IL‐13 (C) or 50 ng/ml IL‐4 (D) during differentiation of NHEKs in 1 mM calcium medium for 7 days. The data shown are representative of three independent experiments from three different donors. mRNA, messenger RNA; NHEK, normal human epidermal keratinocyte; siRNA, small interfering RNA

A growing body of studies have demonstrated that abundant IL‐13, a representative cytokine constituting type 2 inflammatory environment, derived from ILC2 or Th2 plays a pivotal role in the pathogenesis of AD.^27^ On the contrary, accumulating evidence has demonstrated disturbed keratinocyte differentiation in AD.^28^, ^29^ Therefore, we investigated how IL‐13 affects ΔNp63‐mediated keratinocyte differentiation. We treated NHEKs with IL‐13. Morphologically, IL‐13‐stimulated NHEKs showed a smaller and tightly packed polygonal shape compared with control NHEKs (Figure 3A). Next, we investigated *TP63* (ΔNp63) gene expression in response to IL‐13 at various stages of differentiation of NHEKs. Of interest, the gene expression of *TP63* (ΔNp63) was slightly, but significantly, upregulated at all stages of differentiation (Figure 3B). IL‐13 exposure during the 7 days of differentiation inhibited attenuation of ΔNp63 expression, which was confirmed by Western blot analysis (Figure 3C). IL‐4, another type 2 cytokine sharing IL‐4Rα, also inhibited the downregulation of ΔNp63 (Figure 3D). However, transient IL‐13 stimulation at the “pre‐differentiated” or “differentiated” stage did not increase protein expression of ΔNp63 (Figure S2A,B). IL‐22, which is also considered to contribute to AD pathophysiology, did not affect gene or protein expression levels of ΔNp63 (Figure S3A,B).

### 3.4 IL‐13 exposure increased the thickness of 3D‐cultured human NHEKs

**Figure 4:**
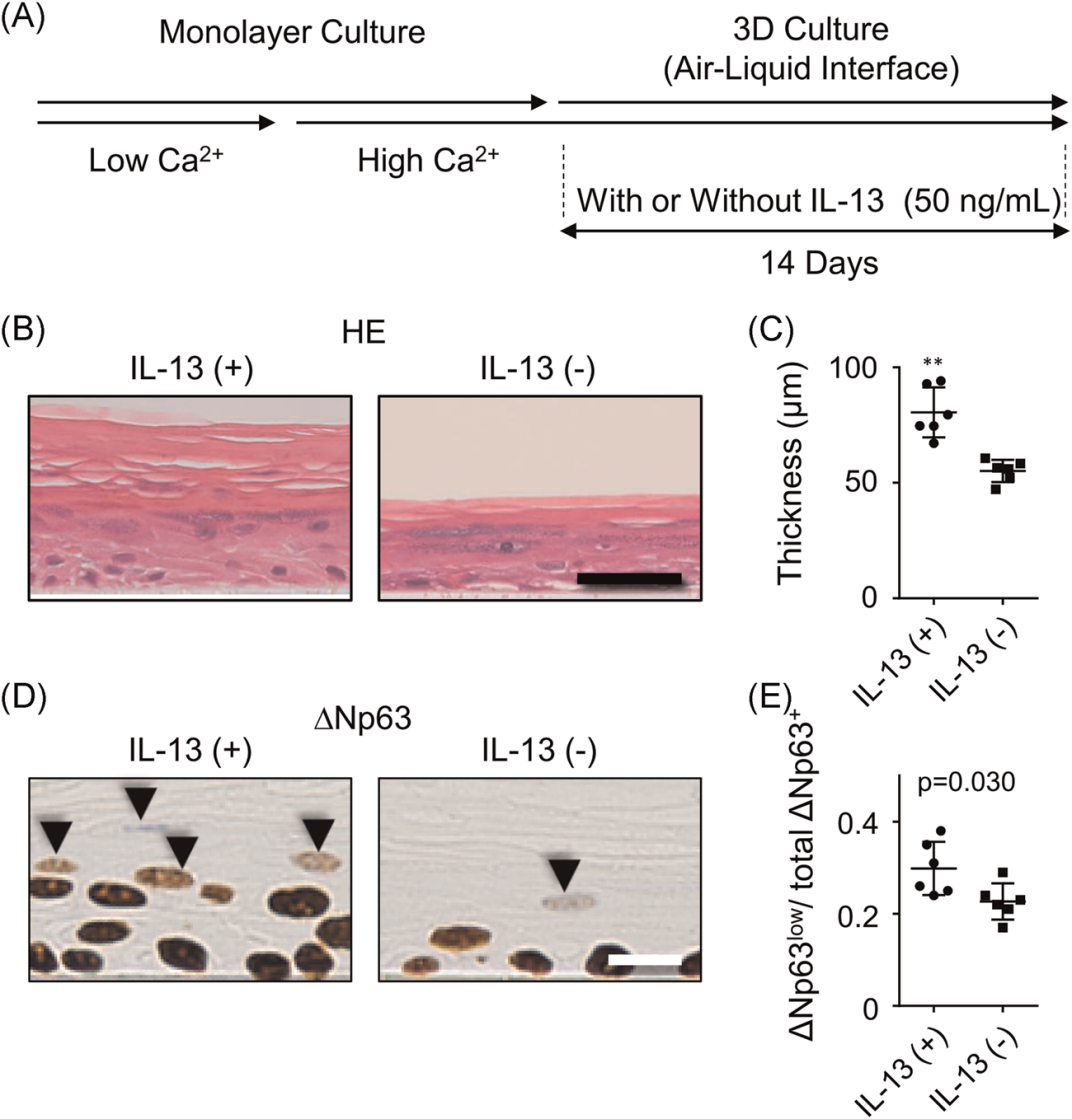
IL‐13 affects the development of 3D‐cultured NHEKs. (A) Schematic diagram of the generation of 3D‐cultured NHEKs using air–liquid interface culture. (B) Hematoxylin and eosin staining of 3D‐cultured keratinocytes, with or without IL‐13, embedded in paraffin and cut perpendicularly. Scale bar = 50 μm. (C) Thickness of 3D‐cultured keratinocytes with or without IL‐13 stimulation. **p < .01. (D) Immunohistochemical staining of ΔNp63 in 3D‐cultured keratinocytes. Arrowheads indicate ΔNp63^low^ cells. Scale bar = 10 μm. (E) Dot plot representing the ratio of ΔNp63^low^ to total ΔNp63^+^ keratinocytes per high‐power field, with or without IL‐13 stimulation. (C, E) Data from six replicative wells under each condition. The data shown are representative of three independent experiments from three different donors. NHEK, normal human epidermal keratinocyte; HE, hematoxylin and eosin stain [Color figure can be viewed at wileyonlinelibrary.com]

To investigate the morphological influence of prolonged stimulation with IL‐13, we stimulated the 3D NHEK culture with IL‐13 (Figure 4A). Of interest, IL‐13‐exposed NHEK culture was statistically significantly thicker compared with the control (Figure 4B,C). Both keratinized and non‐keratinized NHEK layers were thickened (Figure S4A,B). To investigate proliferation potency of NHEK, we performed Ki‐67 expression. While Ki‐67 expression was limited in the basal layer of the control group, in IL‐13‐stimulated condition, parabasal NHEK also expressed Ki‐67 (Figure S4C). The positive rate of Ki‐67 in NHEK was significantly increased in IL‐13‐exposed group (Figure S4D). Similarly, IL‐22‐exposed NHEKs were also hypertrophic (Figure S3C–E). However, although IL‐13‐stimulated NHEKs preserved the granular layer, this layer was lost in IL‐22‐stimulated NHEKs. In addition, IL‐22‐exposed NHEKs showed parakeratosis.

Next, we investigated ΔNp63 expression in the 3D‐cultured human NHEKs with or without IL‐13. Signal intensity of ΔNp63 in the basal layer did not differ between groups. However, we found increased number of decreased but still preserved ΔNp63 expression (ΔNp63^low^ cell in Figure 4D) in the nucleus of IL‐13‐stimulated NHEKs in the upper layer of stratification, where ΔNp63 expression should have ceased and the nuclei of keratinocytes should have degraded under normal conditions. If there are more cells in the field, then accordingly there are more ΔNp63^low^ cells. Therefore, we investigated the ratio of ΔNp63^low^ nuclei to total number of ΔNp63^+^ nuclei, which was significantly higher in IL‐13 stimulation group (Figure 4E).

### 3.5 IL‐13 modulated the expression of ∆Np63‐controlled barrier and inflammation‐related proteins

**Figure 5:**
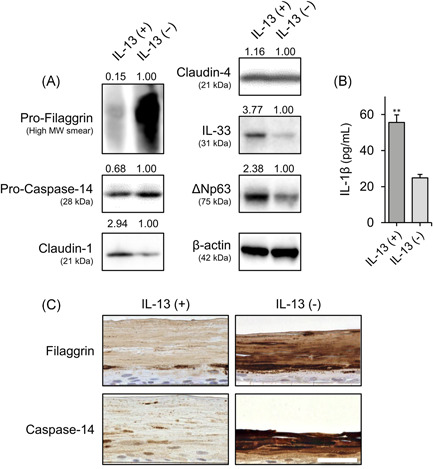
IL‐13 stimulation alters the expression levels of (pro‐)filaggrin, (pro‐)caspase‐14, IL‐33, and IL‐1β in a manner similar to that of ΔNp63. (A) Changes in pro‐filaggrin, pro‐caspase‐14, claudin‐1, claudin‐4, and IL‐33 protein levels in response to stimulation with 50 ng/ml IL‐13 during differentiation of NHEKs in medium containing 1 mM calcium for 7 days. (B) Protein levels of IL‐1β in the culture supernatant of NHEKs with or without 50 ng/ml IL‐13 following poly (I:C) stimulation. **p < .01. (C) Immunohistochemical analysis of filaggrin and caspase‐14. Scale bar = 50 μm. The data shown are representative of three independent experiments from three different donors. NHEK, normal human epidermal keratinocyte [Color figure can be viewed at wileyonlinelibrary.com]

We investigated how IL‐13 exposure affects the ∆Np63‐controlled barrier and inflammation‐related molecules during keratinocyte differentiation. As expected, in Western blot analysis, expression levels of filaggrin, caspase‐14 were lower, and IL‐33 expression level was higher in the IL‐13‐stimulated group compared with the control group (Figure 5A). Furthermore, release of IL‐1β was increased in response to IL‐13 after stimulation with poly (I:C) (Figure 5B). However, claudin‐1 expression was increased in response to IL‐13 exposure (Figure 5A). In addition, IL‐13 stimulation did not alter claudin‐4 expression (Figure 5A). Next, we confirmed that expression of filaggrin and caspase‐14 was decreased by IL‐13 exposure in the 3D‐cultured NHEKs. The expression patterns of filaggrin and caspase‐14 in control groups were similar to those seen in human epidermis. Levels of these proteins were decreased in the IL‐13‐exposed keratinocytes (Figure 5C).

## 4 DISCUSSION

**Figure 6:**
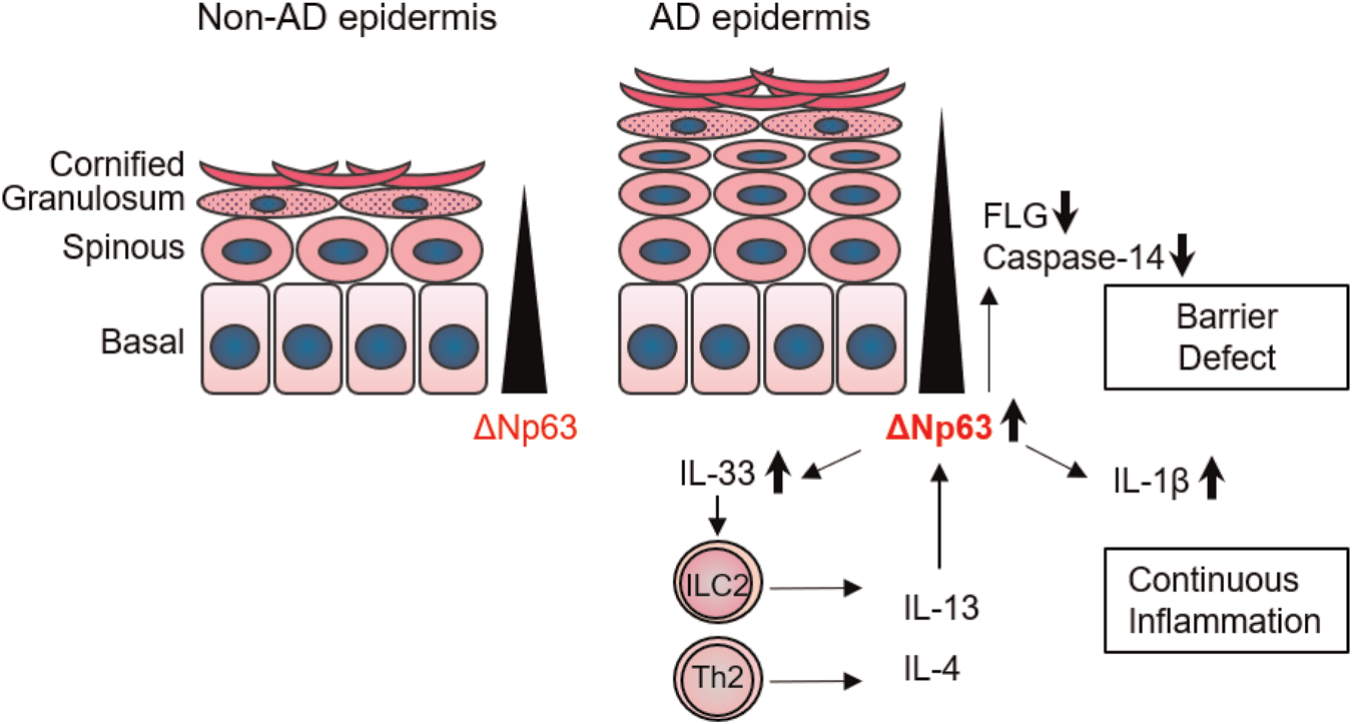
Integrated barrier and immune regulation by ΔNp63 in human NHEKs: schematic diagram representing the findings and conclusions of this study. IL‐13 exposure inhibits keratinocyte differentiation by interrupting ΔNp63 downregulation. Morphologically, epidermal thickness is increased. Decreased filaggrin and caspase‐14 expression further disturb epidermal barrier integrity, whereas increased IL‐1β and IL‐33 enhance inflammation. In addition, IL‐33 stimulates Th2 and ILC2 activity, which are the principal sources of IL‐13. This IL‐13‐ΔNp63 axis may constitute a vicious cycle, partly explaining the chronicity of atopic dermatitis. Note ΔNp63 independent pathway can be also involved. AD, atopic dermatitis; NHEK, normal human epidermal keratinocyte [Color figure can be viewed at wileyonlinelibrary.com]

In this study, we have shown that AD‐related barrier molecules are expressed differently at each stage of differentiation in epidermal keratinocytes. Comprehensive gene expression analysis revealed that ΔNp63, a major regulator of epidermal differentiation, modulated AD‐related barrier and inflammatory molecules. In addition, ΔNp63 expression was affected by IL‐4/IL‐13. Figure 6 presents a schematic diagram summarizing the results of the current study.

In the process of epidermal differentiation, the expression level of ΔNp63 is gradually attenuated. We have shown that IL‐4/IL‐13 interferes with this process. This has been also shown in a recent study by Brauweiler et al.^30^ Given that recent studies have revealed that aberrant keratinocyte differentiation underlies the pathophysiology of AD,^28^, ^29^ the IL‐13–ΔNp63 axis would have an important role in the dysregulated keratinocyte phenotype in AD. We observed that IL‐13 stimulation for 24 h at any stage of differentiation (pre‐differentiated, differentiating, and differentiated stages) slightly upregulated the ΔNp63 gene expression level. However, IL‐13 stimulation could not upregulate the ΔNp63 protein level at the pre‐differentiated or differentiated stage. However, continuous IL‐13 exposure in differentiating keratinocytes inhibited the downregulation of ΔNp63. These findings indicate that IL‐13 interferes with keratinocyte differentiation rather than inducing dedifferentiation.

It was recently reported that dupilumab treatment induced a strong increase in epidermal differentiation markers in the clinical setting.^31^ This may support the clinical relevance of the present study. Type 2 inflammation contributes to the wound healing process.^32^ Considering the role of ΔNp63 as a key regulator of keratinocyte stemness,^33^ it is reasonable that keratinocytes exposed to IL‐13 deviate from differentiation by preserving ΔNp63 expression. This process is assumed to contribute physiologically to epidermal regeneration. Indeed, in 3D‐cultured keratinocyte, IL‐13 stimulation thickened both keratinized and non‐keratinized keratinocyte layers together with increased expression of Ki‐67, a proliferation marker. In the context of AD, the IL‐13–ΔNp63 axis modulated some AD‐related barrier and inflammatory molecules, although further investigation for direct effect of ΔNp63 upregulation on these molecules after IL‐13 treatment is still required. AD is not a single disease entity but an aggregate of different endotypes.^34^ Distinctive endotypes are dependent on ethnic factors or stage of growth. However, dysregulated epidermal barrier and type 2 immune polarization are considered to commonly underlie all types of AD. The expression level of the barrier‐related proteins filaggrin and caspase‐14 were decreased in response to IL‐13, which is consistent with previous findings.^35^, ^36^ The IL‐13–ΔNp63 axis would be involved in these processes. On the contrary, the IL‐13–ΔNp63 axis also enhanced IL‐1β and IL‐33 production. Therefore, we propose that IL‐33 would further enhance type 2 inflammation by activating ILC2 and Th2, leading to a vicious cycle.^13^, ^37^ To date, although barrier disruption and overactivation of immunity in AD keratinocytes have been identified individually, our results indicate that the ΔNp63 pathway at least partially integrates these keratinocyte features. Indeed, we have shown the increased ΔNp63^low^ cells in atopic skin tissue in our previous report.^19^ Decreased expression of ΔNp63 would reflect blockade of keratinocyte differentiation. We propose, however, not only IL‐4/IL‐13 but many other factors should also affect ΔNp63 expression in atopic skin.

Although the important tight junction barrier‐related proteins claudin‐1 and claudin‐4 were also modulated by ΔNp63, IL‐13 did not decrease their expression at the protein level. However, ILC2 disturbed keratinocyte barrier integrity via IL‐13. The transcription levels of *CLDN1* and *CLDN4* were decreased in this setting. It was previously reported that the protein level of claudin‐1 was increased in response to IL‐4/IL‐13. Given that tight junction‐related proteins should be appropriately assembled into the tight junction structure, protein expression level alone is not always correlated with functional barrier integrity. Our findings indicate the further existence of important mechanisms in addition to the IL‐13–ΔNp63 axis in the pathogenesis of AD. We previously demonstrated that the expression of TSLP, another cardinal epithelial cell‐derived cytokine inducing type 2 inflammation, was regulated by ΔNp73.^20^ Although IL‐13 enhanced TSLP production in keratinocytes,^38^ *TP63* (ΔNp63) siRNA did not reproducibly modulate TSLP expression. Given that p53 family proteins interact and/or inhibit each other in a complex manner, IL‐13 may influence the transcriptional regulatory network of p53 family molecules. In addition, during keratinocyte differentiation, other transcription factors, including MafB and/or GATA3, are reported to have a role.^39^, ^40^, ^41^ Of note, ΔNp63 and other keratinocyte differentiation‐related transcription factors respond to various intrinsic and extrinsic exposomes other than IL‐4/IL‐13.^42^ Other differentiation‐related transcription factors affected by exposomes may also play a role in the behavior of keratinocytes.

Notably, ΔNp63 is expressed in the epithelium of the affected organ in other types of type 2 inflammation‐related allergic disease, including bronchial asthma, allergic rhinitis or conjunctivitis, and eosinophilic esophagitis.^21^, ^43^, ^44^, ^45^ In particular, esophageal squamous epithelium exposed to IL‐13 behaved in an opposite manner to ΔNp63‐knockdown keratinocytes in our experiment for some genes expression including *SERPINB3*, *SERPINB4*, or *DAPL1*.^46^ These findings imply that the IL‐13–ΔNp63 axis commonly underlies type 2 allergic inflammation in multiple organs. Furthermore, ΔNp63 expression in the keratinocyte is altered with aging; the elderly express less ΔNp63 in keratinocytes.^47^ Indeed, a previous study revealed that *FLG* expression, which is modulated by ΔNp63, increases with age.^48^ In addition, some children outgrow atopic disease. These results suggest that an aging‐associated decrease in ΔNp63 may underlie epidermal maturation and the ability to outgrow AD.

Although we did not investigate the keratinocyte phenotype in detail in this study, we found that IL‐22 has a role. We also found differential morphological alterations in response to IL‐13 and IL‐22 exposure, whereas exposure to either of these cytokines caused thickening of the 3D‐cultured keratinocyte. Nevertheless, IL‐22 did not change the expression of ΔNp63. IL‐22 has a role in certain endotypes of AD.^49^, ^50^ Together with the IL‐13–ΔNp63 axis, the functional influence of IL‐22 on keratinocytes contributes to the epidermal inflammatory microenvironment of AD.

In conclusion, we identified the potential functional significance of the IL‐13–ΔNp63 axis in barrier‐ and inflammation‐related function of keratinocytes. Although additional investigation of the direct effect of ΔNp63 upregulation on these molecules after IL‐13 treatment is still elusive, our findings suggest that the IL‐13–ΔNp63 axis integrates two major factors of AD pathogenesis: epidermal barrier dysregulation and increased cytokine production in keratinocytes. Furthermore, the IL‐13–ΔNp63 axis might be crucially involved in the vicious cycle of type 2 inflammation. p63/p73 is commonly expressed in epithelial cells in organs involved in atopic diseases. p53‐related transcription factors may universally modulate the local inflammatory microenvironment in atopic diseases. We believe that further investigation of the behaviors of epithelial cells regulated by p53 homologs would provide further understanding as well as novel therapeutic and efficient preventive strategies for type 2 inflammation‐associated allergic diseases.

## CONFLICT OF INTERESTS

The authors declare that there are no conflict of interests.

## AUTHOR CONTRIBUTIONS

Terufumi Kubo planned and performed experiment. Sayuri Sato, Tokimasa Hida, and Tomoyuki Minowa contributed to sample preparation. Terufumi Kubo, Sayuri Sato, Tokimasa Hida, and Tomoyuki Minowa wrote and edited manuscript. Yoshihiko Hirohashi, Tomohide Tsukahara, Takayuki Kanaseki, and Kenji Murata conceived of the study and participated in the design, and the preparation of the manuscript. Hisashi Uhara and Toshihiko Torigoe supervised the project. All authors provided critical feedback and helped shape the research, analysis and manuscript. All authors read and approved the final manuscript.

## Supporting information

Supplementary information.

Supplementary information.

Supplementary information.

Supplementary information.

Supplementary information.

Supplementary information.

Supplementary information.

## Data Availability

The data that support the findings of this study are available from the corresponding author upon reasonable request.
