# Supplementary figures and images for: IL‐13 modulates ∆Np63 levels causing altered expression of barrier‐ and inflammation‐related molecules in human keratinocytes: A possible explanation for chronicity of atopic dermatitis
Source: Immun Inflamm Dis. 2021 Apr 1;9(3):734–45. doi: 10.1002/iid3.427 (PMC8342210; doi:10.1002/iid3.427)

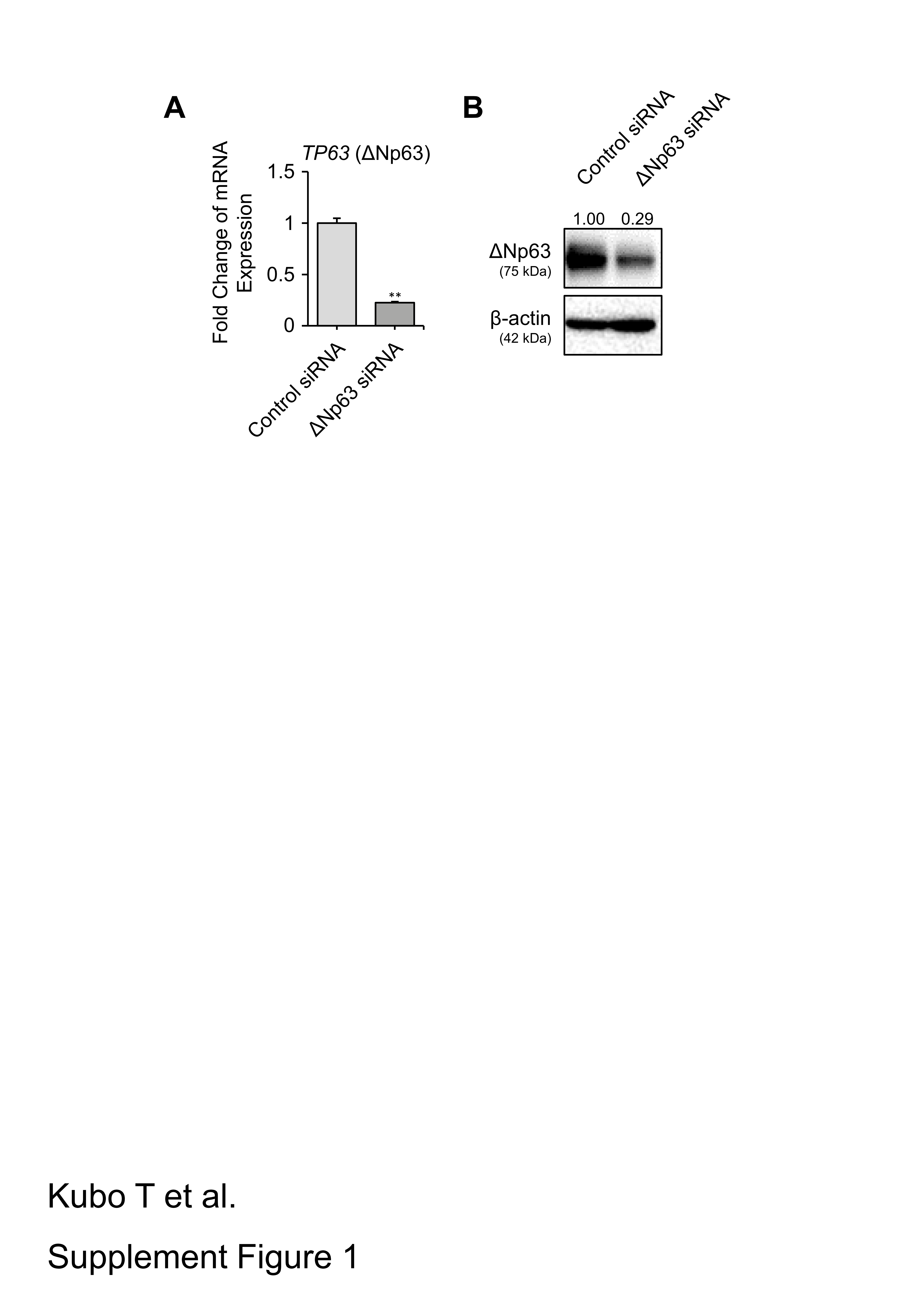

Supplement: Supplementary file 1 — Supplementary information. [file IID3-9-734-s001.tif]

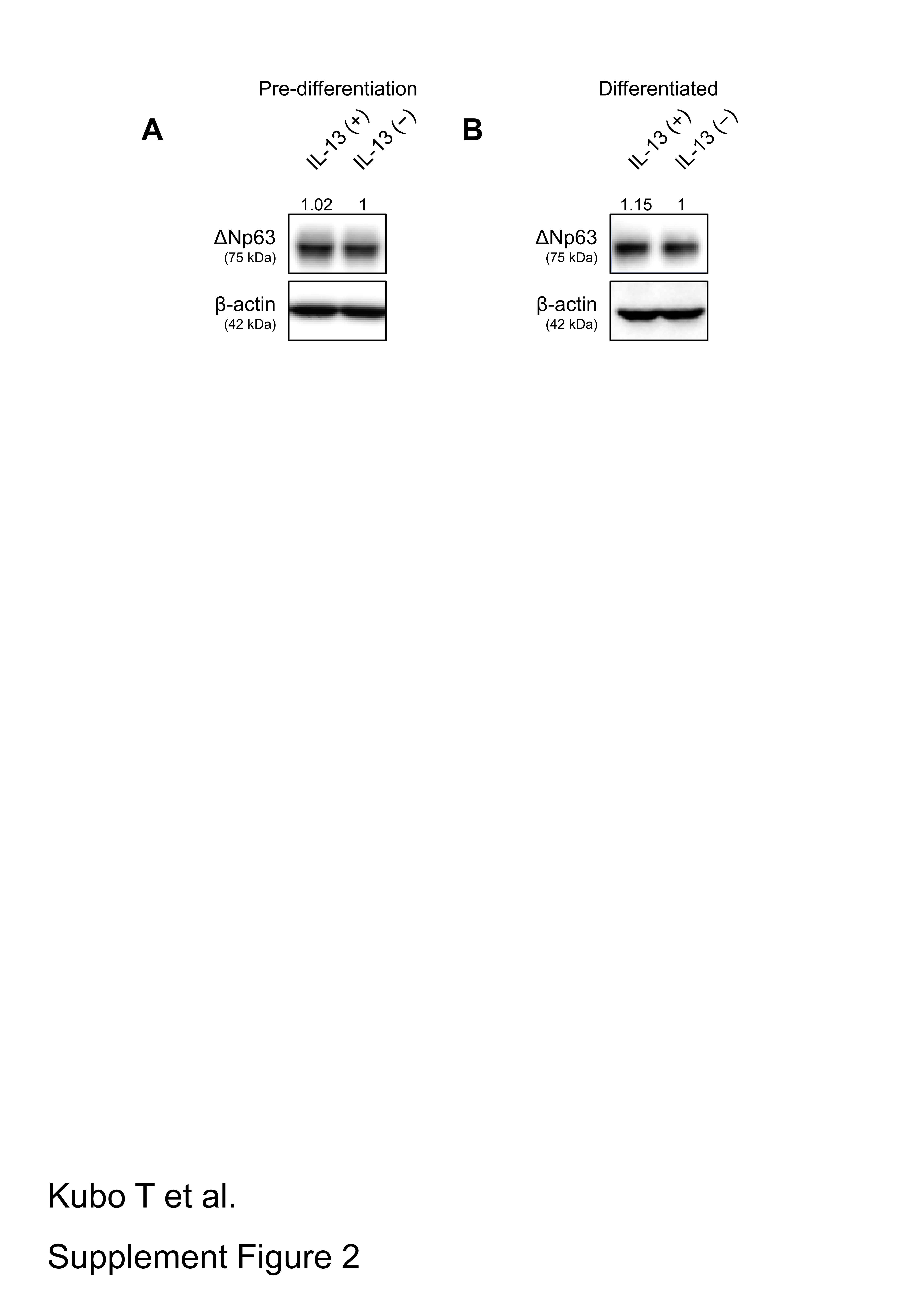

Supplement: Supplementary file 2 — Supplementary information. [file IID3-9-734-s002.tif]

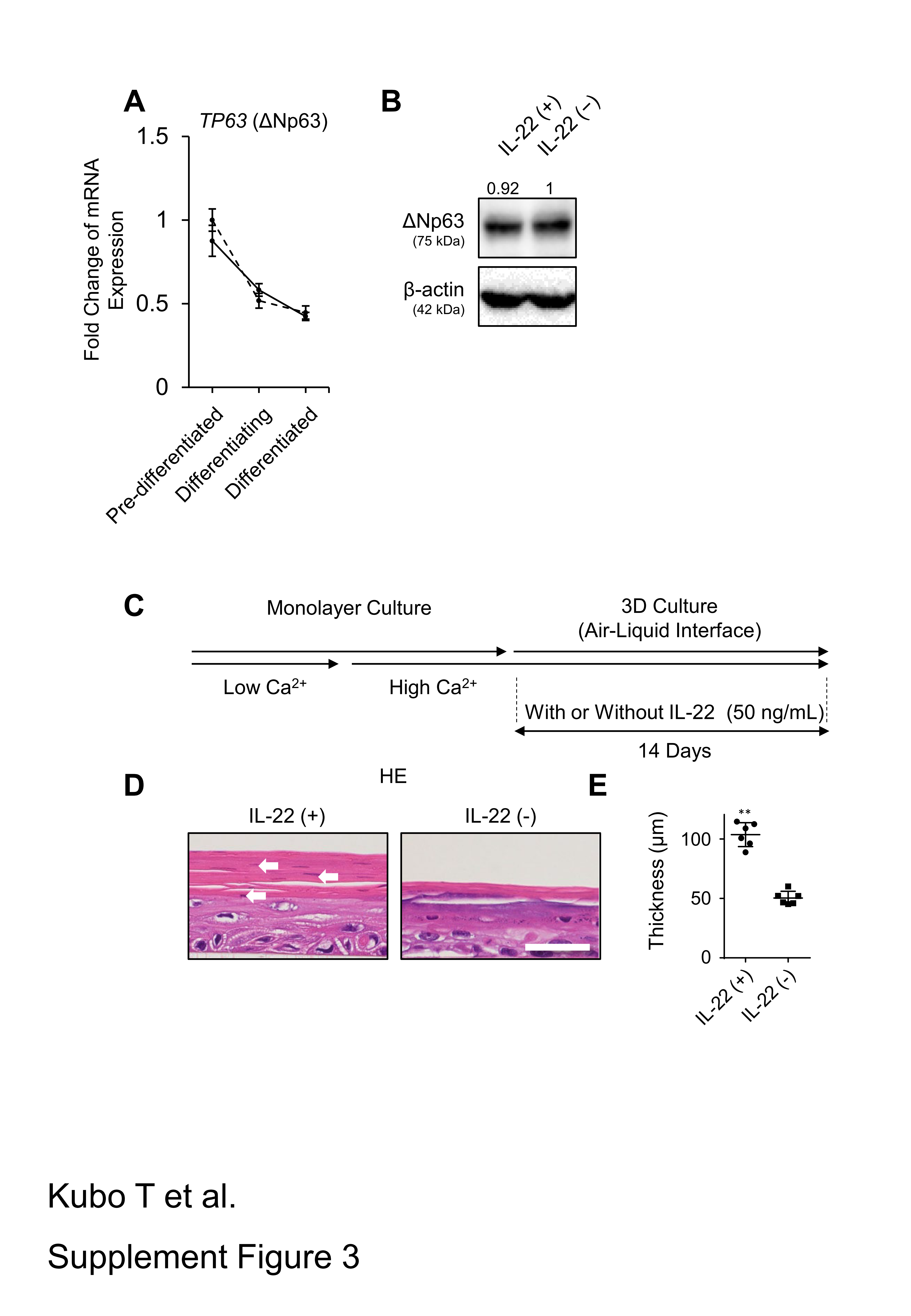

Supplement: Supplementary file 3 — Supplementary information. [file IID3-9-734-s004.tif]

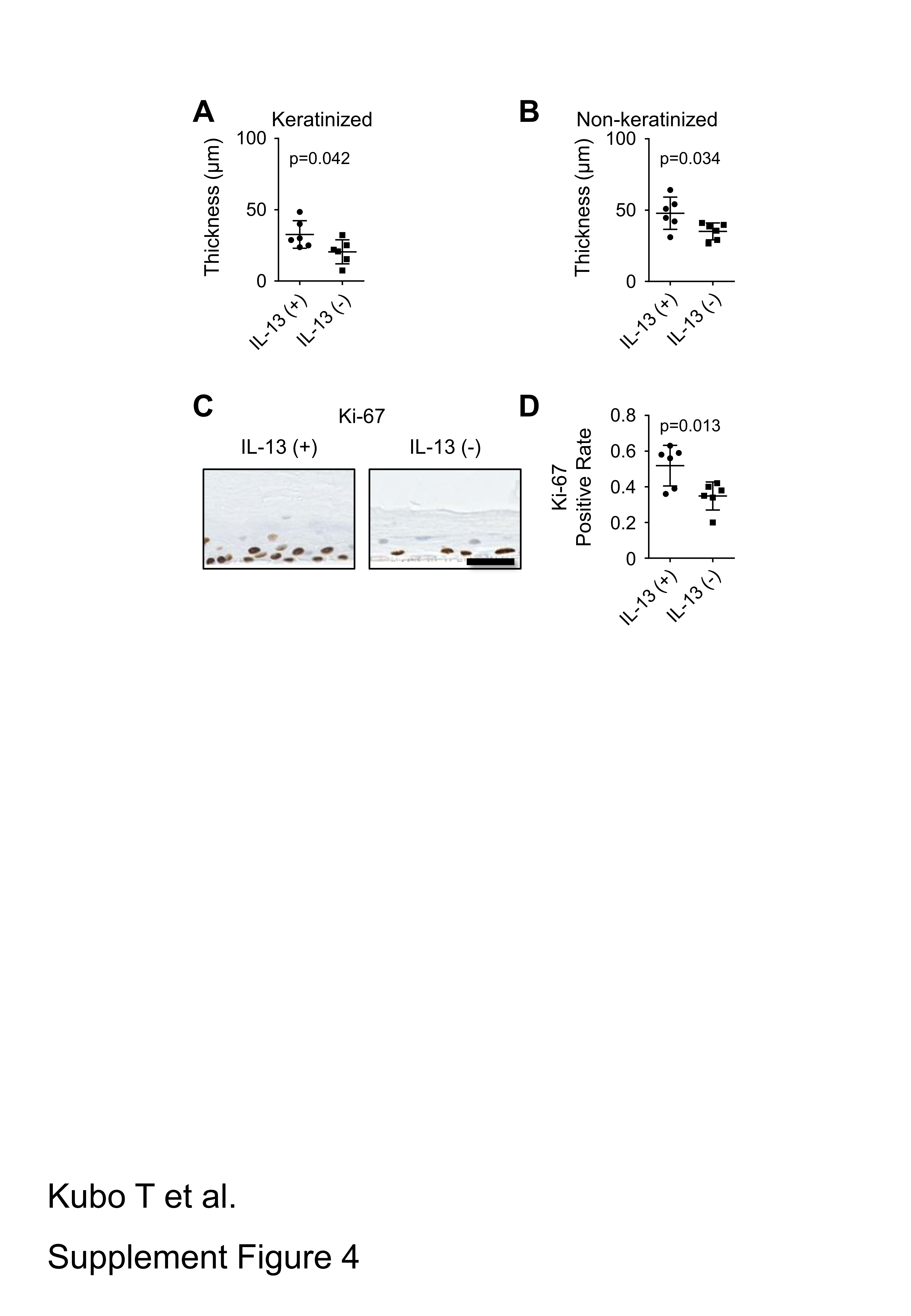

Supplement: Supplementary file 4 — Supplementary information. [file IID3-9-734-s003.tif]
