# Supplementary material for: IL‐13 modulates ∆Np63 levels causing altered expression of barrier‐ and inflammation‐related molecules in human keratinocytes: A possible explanation for chronicity of atopic dermatitis
Source: Immun Inflamm Dis. 2021 Apr 1;9(3):734–45. doi: 10.1002/iid3.427 (PMC8342210; doi:10.1002/iid3.427)
Supplement: Supplementary file 5 — Supplementary information. [file IID3-9-734-s005.docx]

| Gene name | Forward sequence 5′-3′ | Reverse sequence 5′-3′ |
| --- | --- | --- |
| EF1α | CTGAACCATCCAGGCCAAAT | GCCGTGTGGCAATCCAAT |
| ΔNp63 | GGAAAACAATGCCCAGACTC | TGTTATAGGGACTGGTGGAC |
| FLG | CAAATCCTGAAGAATCCAGATGAC | TGCTTGAGCCAACTTGAATACC |
| CASP14 | TGCACGTTTATTCCACGGTA | TGCTTTGGATTTCAGGGTTC |
| CLDN1 | CAGTCAATGCCAGGTACGAATTT | AAGTAGGGCACCTCCCAGAAG |
| CLDN4 | TGTACCAACTGCCTGGAGGAT | GACACCGGCACTATCACCATAA |
| IL1B | ATGATGGCTTATTACAGTGGCAA | GTCGGAGATTCGTAGCTGGA |
| IL33 | GTGACGGTGTTGATGGTAAGAT | AGCTCCACAGAGTGTTCCTTG |

**Supplemental Table 1 Primer sequences for quantitative PCR.**
