# Supplementary material for: IL‐13 modulates ∆Np63 levels causing altered expression of barrier‐ and inflammation‐related molecules in human keratinocytes: A possible explanation for chronicity of atopic dermatitis
Source: Immun Inflamm Dis. 2021 Apr 1;9(3):734–45. doi: 10.1002/iid3.427 (PMC8342210; doi:10.1002/iid3.427)
Supplement: Supplementary file 7 — Supplementary information. [file IID3-9-734-s007.docx]

Supplemental Figure 1 **Knockdown of ΔNp63 by siRNA in NHEKs.**

(A) Quantitative PCR and (B) immunoblot demonstrating levels of ΔNp63 in siRNA for ΔNp63 or control siRNA transfected NHEKs. (A, B) Cells were harvested 72▒h after transfection. **P▒<▒0.01. The data shown are representative of three independent experiments from three different donors.

Supplemental Figure 2 **Influence of IL-13 stimulation on ΔNp63 expression in NHEKs at different stages of differentiation.**

(A) NHEKs at the pre-differentiation stage were stimulated with 50▒ng/ml IL-13 for 24▒h. (B) Differentiated NHEKs were cultured in 1▒mM calcium medium for 7 days, and then stimulated with 50▒ng/ml IL-13 for 24▒h. The data shown are representative of three independent experiments from three different donors.

Supplemental Figure 3 **IL-22 affects the development of 3D cultured human NHEKs, but not ΔNp63 expression.**

(A) ΔNp63 gene expression in response to 50▒ng/ml IL-22 at various stages of differentiation of NHEKs. (B) Expression of ΔNp63 protein in response to 50▒ng/ml IL-22 during differentiation of NHEKs in 1▒mM calcium medium for 7 days. (C) Schematic diagram of the generation of 3D culture of keratinocytes in air–liquid interface culture. (D) Hematoxylin and eosin staining of 3D cultured NHEKs, with or without IL-22, embedded in paraffin and cut perpendicularly. Arrows indicate nuclear remnant of parakeratosis. Bar▒=▒50 μm. (E) Thickness of 3D cultured NHEKs with or without IL-22 stimulation. **P▒<▒0.01. The data shown are representative of three independent experiments from three different donors.

Supplemental Figure 4 **IL-13 affects the proliferation of 3D cultured human NHEKs.**

(A, B) Thickness of keratinized (A) and non-keratinized (B) keratinocyte layers with or without IL-13 stimulation. (C) Immunohistochemical staining of Ki-67, a marker indicating proliferation, in 3D cultured keratinocytes. Bar▒=▒50 μm. (D) Ki-67 positive rate is shown. The data shown are representative of three independent experiments from three different donors.
